# Control of virulence gene transcription by indirect readout in *Vibrio cholerae* and *Salmonella enterica* serovar Typhimurium

**DOI:** 10.1111/1462-2920.13838

**Published:** 2017-07-24

**Authors:** Charles J. Dorman, Matthew J. Dorman

**Affiliations:** ^1^ Department of Microbiology Moyne Institute of Preventive Medicine, Trinity College Dublin Dublin Ireland; ^2^ Wellcome Trust Sanger Institute, Wellcome Genome Campus Hinxton Cambridgeshire CB10 1SA UK

## Abstract

Indirect readout mechanisms of transcription control rely on the recognition of DNA shape by transcription factors (TFs). TFs may also employ a direct readout mechanism that involves the reading of the base sequence in the DNA major groove at the binding site. TFs with winged helix–turn–helix (wHTH) motifs use an alpha helix to read the base sequence in the major groove while inserting a beta sheet ‘wing’ into the adjacent minor groove. Such wHTH proteins are important regulators of virulence gene transcription in many pathogens; they also control housekeeping genes. This article considers the cases of the non‐invasive Gram‐negative pathogen *Vibrio cholerae* and the invasive pathogen *Salmonella enterica* serovar Typhimurium. Both possess clusters of A + T‐rich horizontally acquired virulence genes that are silenced by the nucleoid‐associated protein H‐NS and regulated positively or negatively by wHTH TFs: for example, ToxR and LeuO in *V. cholerae*; HilA, LeuO, SlyA and OmpR in *S*. Typhimurium. Because of their relatively relaxed base sequence requirements for target recognition, indirect readout mechanisms have the potential to engage regulatory proteins with many more targets than might be the case using direct readout, making indirect readout an important, yet often ignored, contributor to the expression of pathogenic phenotypes.

## Introduction

Gram‐negative enteric pathogens have acquired many of the virulence genes used for infection of their hosts through horizontal gene transfer (Ochman *et al*., 2000; Dorman, 2009; Bliven and Maurelli, 2016; Navarre, 2016). The expression of these genes is usually restricted to sites on or in the host where their products can contribute to a specific stage of the infection process. Signals associated with those sites are detected by the bacterium and transduced to the virulence genes (Skorupski and Taylor, 1997; Rhen and Dorman, 2005; Erhardt and Dersch, 2015; Ayala *et al*., 2017). Gene activation at the level of transcription typically involves DNA‐binding proteins that recruit and/or activate RNA polymerase at specific promoters and/or remove factors that silence transcription (Stoebel *et al*., 2008; Browning and Busby, 2016).

The evidence that lateral transfer has played a role in the acquisition of important virulence genes has come, in part, from an analysis of the base composition of the genes. Virulence genes have a higher A + T base content than the average for the core genome. They are frequently grouped in clusters called pathogenicity islands that possess features normally associated with mobile genetic elements (Hazen *et al*., 2010; Syvanen, 2012; Rodriguez‐Valera *et al*., 2016). A combination of regulatory proteins encoded by the core and the horizontally acquired genome controls virulence gene transcription (Rhen and Dorman, 2005). Further evidence has come from observing horizontal transfer taking place in contemporary pathogens: for example, the transduction of the genes encoding cholera toxin by the CTXΦ filamentous bacteriophage in *Vibrio cholerae* (Waldor and Mekalanos, 1996).

Transcription silencing by the H‐NS nucleoid‐associated protein is a common feature of virulence genes in enteric pathogens (Porter and Dorman, 1994; Hromockyj *et al*., 2002; Beloin and Dorman, 2003; Lucchini *et al*., 2006; Baños *et al*., 2008; Zwir *et al*., 2014; Dorman, 2015; Hüttener *et al*., 2015; Prieto *et al*., 2016). This protein targets genes with a high A + T base content and prevents their transcription by RNA polymerase (Dame *et al*., 2001, 2002; Beloin and Dorman, 2003; Dorman, 2004; Lucchini *et al*., 2006). The relief of H‐NS silencing is achieved by an impressively wide range of mechanisms, many of which rely on DNA binding proteins that target H‐NS‐silenced promoters and either remove H‐NS or remodel the nucleoprotein complex in ways that facilitate the activation of gene expression (Prosseda *et al*., 2004; Stoebel *et al*., 2008).

It has been proposed that the transcription silencing activity of H‐NS assists in the evolution of pathogens by allowing them to acquire new genes and then to incorporate them into the genome at a regulatory level as well as integrating them physically (Dorman, 2007; Ali *et al*., 2014). Silencing prevents the inappropriate expression of new genetic information that could, if not properly controlled, compromise the competitive fitness of the bacterium (Lucchini *et al*., 2006; Dorman, 2007; Navarre, 2016). The requirement by H‐NS for an A + T‐rich DNA substrate on which to construct its silencing complex provides the basis for a crude‐yet‐effective regulatory switch. In this switch, H‐NS imposes transcriptional silence. For the switch to be reversed, an H‐NS antagonist with similar requirements in its binding substrate would be required so that the opposing proteins can encounter one another on the DNA (Stoebel *et al*., 2008; Dorman and Kane, 2009; Kane and Dorman, 2011).

**Figure 1:**
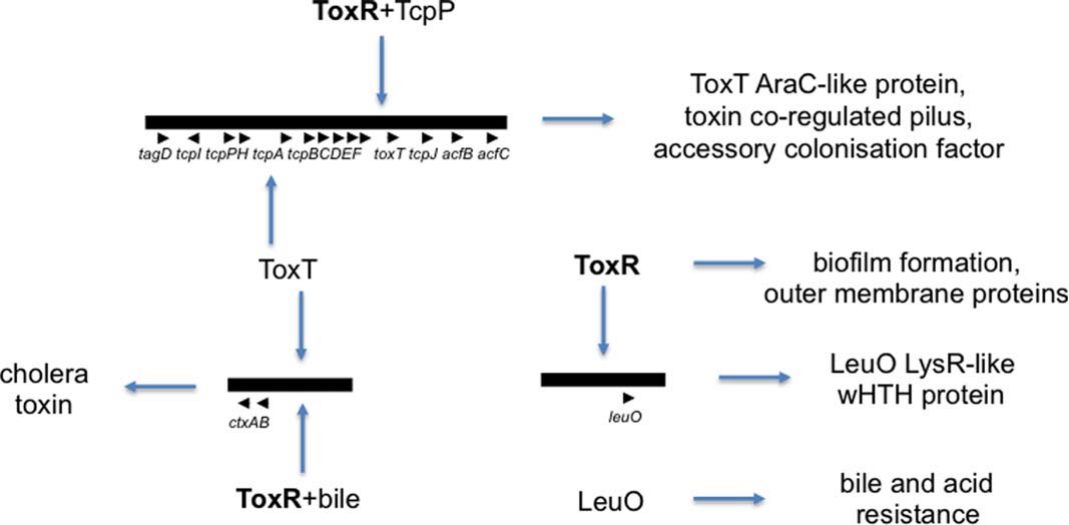
ToxR‐associated virulence gene control circuits in *Vibrio cholerae*. Genes and phenotypes governed by the wHTH TF ToxR (bold type) are shown. ToxR works in association with another wHTH protein, TcpP, to activate transcription of the *toxT* gene. ToxT positively autoregulates *toxT* transcription from an upstream promoter at *tcpA*. The AraC‐like ToxT protein is the principal activator of the cholera toxin operon, *ctxAB*, although ToxR can also activate its transcription when over‐expressed or in the presence of bile. Among the genomic targets of ToxR are genes contributing to biofilm formation; ToxR also activates the expression of the *leuO* gene whose LysR‐like protein product is involved in resistance to bile and acid. Genomic segments are represented by horizontal bars and individual genes by arrowheads (not to scale).

**Figure 2:**
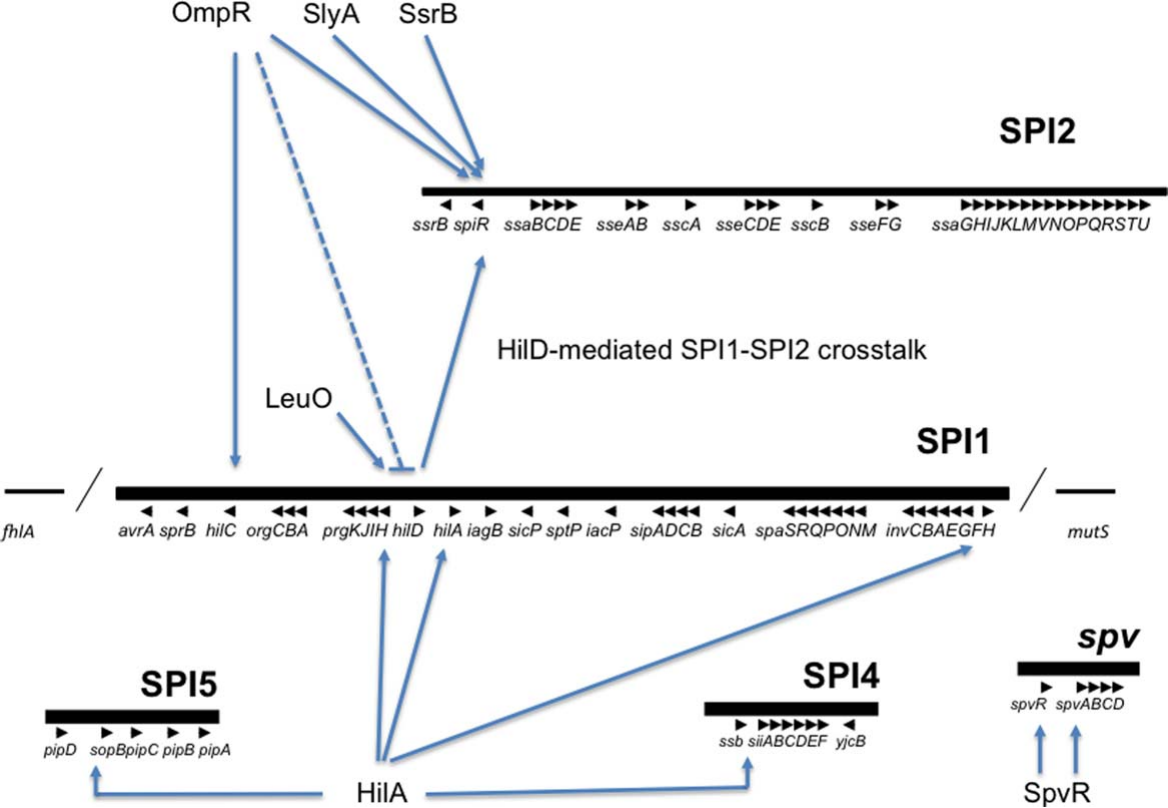
Virulence gene control and wHTH TFs in *Salmonella enterica* serovar Typhimurium. Pathogenicity islands in the chromosome and the *spv* locus on the *Salmonella* Virulence Plasmid are represented by horizontal bars with arrowheads representing individual genes (not to scale). Regulatory inputs by various wHTH TFs mentioned in the text are indicated by arrows (positive inputs) or dotted ‘T’ bars (negative inputs). HilA autoregulates *hilA* transcription in addition to controlling other genes in SPI1, SPI4 and SPI5. HilD is an AraC‐like TF, it regulates *hilA* in association with AraC‐like activators HilC and RstA; SsrB is a member of the NarL/FixJ response regulator subfamily (Carroll *et al*., 2009; Walthers *et al*., 2011).

Here it will be proposed that DNA binding proteins which use a winged helix–turn–helix motif (wHTH) to bind to A + T‐rich DNA are particularly well suited to this task (Brennan, 1993; Schell, 1993). The point will be illustrated with reference to the intensively studied enteric pathogens *Vibrio cholerae* (Fig. 1) and *Salmonella enterica* serovar Typhimurium (Fig. 2). These organisms use very different strategies for infection, yet have similar mechanisms for virulence gene regulation. The similarities include a reliance on wHTH transcription factors to overcome H‐NS‐mediated silencing of A + T‐rich genes that have been acquired by lateral gene transfer (Bajaj *et al*., 1995; Nye *et al*., 2000; Kenney, 2002; Dolan *et al*., 2011; Quinn *et al*., 2014; Kazi *et al*., 2016).

## Direct versus indirect readout in gene regulation

**Figure 3:**
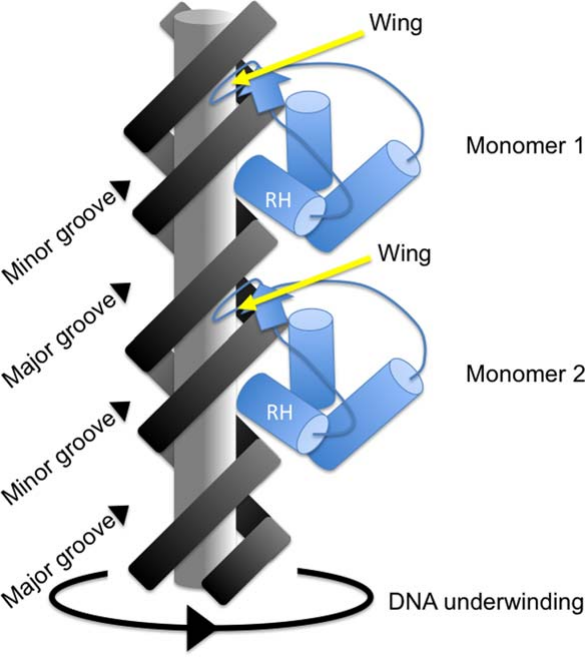
Interaction of a wHTH TF with DNA. A diagram of a vertical segment of B‐DNA is shown with the major and minor grooves labelled. The wHTH motifs of each monomer of a dimeric DNA binding protein are shown in blue. Cylinders represent alpha helices with the recognition helix labelled 'RH' in each case. Yellow arrows point to the wings inserted into the minor groove of the DNA. (Not to scale.) Rotating the DNA in the direction of the curved arrow at the bottom while holding the top of the duplex immobile would mimic the effect of underwinding the DNA as a result of negative supercoiling.

Transcription factors that select their binding sites by detecting specific base sequences are said to use a direct readout mechanism for the regulation of their target genes (Travers, 1997; Mendieta *et al*., 2007). Base sequence information is most readily accessible in the major groove of DNA with transcription factors that use direct readout typically possessing a motif for insertion into that groove (Steffen *et al*., 2002). In the case of proteins that use a helix–turn–helix (HTH) motif, that feature is an alpha helix (Brennan and Matthews, 1989; Harrison and Aggarwal, 1990) (Fig. 3).

Indirect readout refers to regulatory mechanisms where the transcription factor reads the shape of the DNA molecule rather than relying on the base sequence as the main source of information (Rohs *et al*., 2010; Slattery *et al*., 2014; Yang *et al*., 2017). Base content and sequence both affect DNA shape but they work in the context of other influences (Rohs *et al*., 2009). These include changes to shape that arise from modifications to the parameters of DNA topology: linking number, twist and writhe (Bauer *et al*., 1980; Mathelier *et al*., 2016). Other factors that have an impact include intrinsic DNA curvature and variations in DNA flexibility and persistence length (Travers, 1997). In addition to DNA shaping influences that are intrinsic to the polymer, there are also modifications to shape that are imposed by DNA binding proteins: nucleoid‐associated proteins bend, wrap or bridge DNA and most other proteins that bind to DNA will cause some distortion to its topology (Dillon and Dorman, 2010). Molecular crowding within the cytoplasm may also impose change on DNA shape (Zimmerman, 2006) and very localised modifications can be produced by the methylation of particular bases (Casadesús, 2016). This constellation of influences creates the possibility of a wide variety of modifications to local and global DNA shape without the need for any changes to the base sequence of the DNA.

Indirect readout by transcription factors has received a great deal of attention in studies of eukaryotes (Joshi *et al*., 2007; Gordân *et al*., 2013; Abe *et al*., 2015; Mathelier *et al*., 2016; Yang *et al*., 2017). In bacterial systems there has been historically more emphasis on direct readout, perhaps because regulatory interactions that rely on the reading of DNA base sequence have provided important regulatory paradigms (e.g., the Lac repressor, the lambda CI repressor and the cyclic AMP–Crp complex), and the presence of matches to protein‐binding‐site consensus sequences in DNA is easier to study *in silico* (Chen *et al*., 1971; Johnson *et al*., 1978; Steitz *et al*., 1982). Arguably, the difficulty in developing bioinformatic tools that can interrogate protein–DNA interactions guided by DNA shape has caused a significant bottleneck in the development of the field (Chiu *et al*., 2014).

Having a narrow minor groove is an important feature of A + T‐rich DNA; the higher the A + T content, the narrower the groove becomes (Rohs *et al*., 2009). The minor groove width narrows over a three‐fold range as one moves from G + C‐rich to A + T‐rich sequences (Rohs *et al*., 2009). This may have implications for the binding to DNA of proteins that engage the minor groove (Fig. 3). Furthermore, changes to the supercoiling of the DNA alter the minor groove width with compensatory adjustments to the width of the major groove (Vologodskii and Cozzarelli, 1994), introducing another variable to modulate DNA–protein interactions. In this context, it is interesting to note that several gene regulatory proteins that antagonise H‐NS‐mediated silencing in A + T‐rich DNA rely on a wHTH motif to interact with both the major and the minor groove of DNA. Among these are ToxR, the master regulator of virulence gene expression in *Vibrio cholerae* (Fig. 1), HilA, a key regulator of virulence gene expression in the SPI1 pathogenicity island of *Salmonella enterica* serovar Typhimurium, and OmpR, a regulator of housekeeping genes together with SPI1‐ and SPI2‐located virulence genes in *S*. Typhimurium (Fig. 2) (Bajaj *et al*., 1995; Martínez‐Hackert and Stock, 1997). In the case of OmpR, changes to DNA supercoiling alter the affinity of binding of the OmpR protein to its targets *in vivo* and *in vitro* (Cameron and Dorman, 2012; Quinn *et al*., 2014).

## ToxR in *V. cholerae*

*V. cholerae* is a non‐invasive pathogen of humans and has an aquatic reservoir in the environment. Toxigenic strains of *V. cholerae* are the cause of Asiatic cholera, and these express a potent enterotoxin together with secondary virulence factors. Cholera toxin, encoded by the cholera toxin operon *ctxAB*, is produced in the small intestine where it causes a dysregulation of human adenylate cyclase, disturbing intestinal epithelial physiology leading to loss of water and electrolytes accompanied by severe watery diarrhoea that is characteristic of the disease (Robins and Mekalanos, 2014).

The ToxR protein is a cytoplasmic‐membrane‐associated DNA binding protein that controls, *inter alia*, the transcription of the *toxT* gene, a positive regulator of *ctxAB* and several other virulence genes in *V. cholerae* (Fig. 1) (Miller *et al*., 1987; Higgins and DiRita, 1994; Yu and DiRita, 1999). The amino terminal portion of ToxR contains a wHTH motif within a segment that is related to the DNA binding domain of the OmpR protein (Miller *et al*., 1987). ToxR binds to DNA that has a very high A + T content and many of the sites in the *V. cholerae* genome that are bound by ToxR are also targets for the *V. cholerae* orthologue of H‐NS (Kazi *et al*., 2016). This protein, sometimes called VicH, is approximately 50% identical in amino acid sequence to H‐NS from *Escherichia coli* (Tendeng *et al*., 2000; Cerdan *et al*., 2003). VicH can substitute for H‐NS in an *E. coli hns* mutant, showing that the proteins share functional similarity in gene regulation (Tendeng *et al*., 2000). The primary role of the *V. cholerae* ToxR protein is to de‐repress genes that are silenced by H‐NS/VicH; in the absence of the nucleoid‐associated protein, ToxR becomes redundant for *V. cholerae* virulence gene expression (Nye *et al*., 2000; Kazi *et al*., 2016). The H‐NS protein also plays a central role in controlling the wider virulence programme of *V. cholerae* beyond the ToxR regulon (Ayala *et al*., 2015; 2017; Dorman, 2015).

ToxR activation of the *toxT* promoter involves an interaction with a second membrane‐associated wHTH protein, TcpP (Krukonis and DiRita, 2003) (Fig. 1). An unusual role has been proposed for the wing in the wHTH motif of TcpP in promoting protein–protein interaction with ToxR: together the proteins control *toxT* transcription through a division of labour with ToxR acting to overcome H‐NS‐mediated silencing while TcpP recruits RNA polymerase to the promoter without TcpP having to bind to DNA (Krukonis and DiRita, 2003). This represents an interesting example of repurposing of a component in a TF normally associated with DNA binding through partnership with a second protein that provides a DNA binding function (Haas et al., 2015). ToxR seems only to form this partnership with TcpP at a very small subset of ToxR targets in the genome, one of which is the *toxT* promoter (Kazi *et al*., 2016).

## The SPI1‐encoded HilA virulence regulator in *S*. Typhimurium

The HilA protein is encoded by the *hilA* gene in the SPI1 pathogenicity island of *S*. Typhimurium where it regulates the transcription of genes required for invasion of the host epithelium in the small intestine (Fig. 2) (Rhen and Dorman, 2005; Thijs *et al*., 2007). The positively autoregulated *hilA* gene has been acquired through lateral gene transfer and so have its most important target genes (Lucchini *et al*., 2006). The protein binds to A + T‐rich DNA using a wHTH motif to activate SPI1, SPI4 and SPI5 promoters that are subject to H‐NS‐mediated transcription silencing (Ahmer *et al*., 1999; Lostroh *et al*., 2000; Main‐Hester *et al*., 2008). Expression of the *hilA* gene is under multifactorial control. In addition to positive regulation by its own product, *hilA* is silenced by a complex consisting of the Hha and H‐NS nucleoid‐associated proteins (Fahlen *et al*., 2001; Queiroz *et al*., 2011; Ali *et al*., 2013). Transcription of the *hilA* gene is also stimulated by the SPI1‐encoded AraC/XylS‐like regulatory proteins HilC, HilD and RtsA (Eichelberg and Galán, 1999; Lostroh *et al*., 2000; Rhen and Dorman, 2005). These proteins resemble ToxT from *V. cholerae* in that they are members of the AraC‐like TF family and use HTH motifs to bind to A + T‐rich DNA. A further point of similarity concerns the sensitivity of their target genes to changes in DNA supercoiling in *S*. Typhimurium (Cameron and Dorman, 2012), *V. cholerae* (Parsot and Mekalanos, 1992) and other pathogens, such as *Shigella flexneri* (Tobe *et al*., 1995).

## The OmpR wHTH regulatory protein

OmpR is a DNA binding protein of the response regulator family and uses a wHTH motif to bind to its targets in DNA (Martínez‐Hackert and Stock, 1997; Rhee *et al*., 2008). The DNA binding activity of OmpR is triggered by phosphorylation of an aspartic acid at position 55 in its ‘receiver’ domain (Delgado *et al*., 1993). EnvZ is the sensor‐kinase that specifically phosphorylates OmpR (Cai and Inouye, 2002). EnvZ first becomes phosphorylated on a histidine at position 243 within its ‘transmitter’ domain in response to an environmental signal and subsequently transfers the phosphate to OmpR (Foo *et al*., 2015). The OmpR protein can act as a repressor or an activator of transcription; its regulatory mode is dictated by the positions of its binding sites relative to the promoter(s) of the target gene and their relative affinities for OmpR (Pratt *et al*., 1996).

In *E. coli*, osmotic stress is a major signal for the EnvZ‐OmpR phosphorelay but acid pH is also known to be an important signal (Stincone *et al*., 2011). In *S*. Typhimurium, acid pH is particularly important for OmpR activation and for the adaptation of the bacterium to low pH experienced during the stationary phase of the growth cycle (Bang *et al*., 2002). OmpR binds to over 200 genomic targets in *S*. Typhimurium, far more than in *E. coli*, and many of the genes that it regulates have been imported by horizontal gene transfer and play a role in infection (Quinn *et al*., 2014). Among these horizontally acquired genes are those that *S*. Typhimurium uses to adapt to the hostile environment within the vacuole of a macrophage. These genes are located in the SPI2 pathogenicity island and are under the control of the SpiR‐SsrB two‐component regulatory system: SpiR (sometimes called SsrA) is a cytoplasmic membrane located sensor kinase and SsrB is a response regulator (Fig. 2) (Feng *et al*., 2003; Fass and Groisman, 2009). This is an interesting example of the integration of regulatory inputs that are fed through one two‐component system (EnvZ‐OmpR) encoded by the core genome and a second system (SpiR‐SsrB) that is encoded by the horizontally acquired portion of the genome (Rhen and Dorman, 2005). The OmpR protein regulates the transcription of the *spiR* and *ssrB* genes, partly by acting there as a conventional TF and partly by acting as an anti‐repressor that overcomes H‐NS‐mediated transcription silencing (Walthers *et al*., 2011). The OmpR protein also controls the transcription of key regulatory genes in the SPI1 island including *hilC* and *hilD*, allowing OmpR to contribute to the coordination of expression of these major virulence genetic elements (Cameron and Dorman, 2012; Quinn *et al*., 2014).

The OmpR protein is influenced in its binding activity by the topological state of the target DNA. Overall, OmpR will bind more avidly to a DNA sequence in a relaxed template than to the same DNA sequence in a more negatively supercoiled conformation (Cameron and Dorman, 2012; Quinn *et al*., 2014). This allows the DNA to play an allosteric role in determining the binding activity of the protein. Why might this be useful? Negative supercoiling is introduced to DNA by the type II topoisomerase, DNA gyrase (Gellert *et al*., 1976; Higgins *et al*., 1978). It can also be introduced at a local level as a by‐product of the processes of transcription and DNA replication (Liu and Wang, 1987). In the case of gyrase, the enzyme hydrolyses ATP to complete each cycle of DNA underwinding. One of the products of this reaction, ADP, is an inhibitor of the supercoiling activity of gyrase. Thus, shifts in the ratio of the intracellular concentration of ATP to ADP in favour of ADP result in a global shift in DNA topology toward a more relaxed template. This happens when metabolic flux rates in the cell decline, leading to an extension in doubling time and eventually, the onset of stationary phase (Conter *et al*., 1997; Dorman *et al*., 2016; Dorman and Dorman, 2016). Topological shifts in DNA shape throughout the growth cycle present to DNA binding proteins the same sets of binding site sequences in a variety of conformations. This variety may become manifest across different cells in the population, leading to distinct readouts from the same genetic elements. The resulting physiological heterogeneity allows the population to ‘hedge its bets’ regarding the environmental challenges and opportunities with which it may be faced. Although the importance of physiological heterogeneity to overall population fitness is generally understood, the contribution of variable DNA topology to achieving this heterogeneity is less well appreciated (Dorman *et al*., 2016).

## Integrated regulatory circuits and evolution

The OmpR/SsrB example of gene regulation in the *S*. Typhimurium SPI2 pathogenicity island is an excellent illustration of cooperation between gene regulators encoded by the core and ‘accessory’ parts of the genome (Fig. 2). What is it about OmpR that makes it such a useful protein for controlling the expression of so many genes in both parts of the genome? The answer seems to include its tolerance of base sequence variety in the DNA targets to which it binds. These are characterised by a high A + T content and we have already seen that this property is a feature of the horizontally acquired components of genomes in Gram‐negative bacteria, especially enteric pathogens. Thus, OmpR, with its wHTH DNA binding motif, is literally a good fit for laterally acquired genes with this high A + T profile. The same DNA is also an attractive target for the transcription‐silencing H‐NS nucleoid‐associated protein (Lucchini *et al*., 2006; Navarre, 2016). This protein shares with OmpR sensitivity to DNA features other than base sequence: although an optimal sequence for H‐NS binding has been described, its chief characteristics are a high A + T content and intrinsic DNA flexibility (Lang *et al*., 2007). Conflicts between OmpR and H‐NS at the same A + T‐rich DNA targets provide a basis for a simple genetic switch, allowing those environmental signals that flow to OmpR (osmotic and acid stress) to influence transcription by overcoming H‐NS‐mediated repression. By relying on features of DNA other than just the presence of very close matches to a consensus base sequence for binding, wHTH proteins can participate in, and drive, the rapid evolution of control circuits. Because of their relatively relaxed base sequence requirements for target recognition, indirect readout mechanisms have the potential to engage regulatory proteins with many more targets than might be the case using direct readout. Changes to DNA shape brought about through alterations in the parameters of DNA supercoiling (linking number, twist and writhe) in response to fluctuations in metabolic flux create a very dynamic profile of DNA targets. A target that is proficient for protein binding at a given superhelical density (σ) may be much less proficient at another value of σ as is the case for the OmpR protein in *S*. Typhimurium (Cameron and Dorman, 2012; Quinn *et al*., 2014).

## Other wHTH proteins and *V. cholerae* and *S*. Typhimurium pathogenesis

The SlyA protein of *S*. Typhimurium and *E. coli* uses a wHTH motif to bind to DNA and is frequently found to overcome H‐NS‐mediated transcription silencing (Westermark *et al*., 2000; Corbett *et al*., 2007; Lithgow *et al*., 2007; Perez *et al*., 2008; Weatherspoon‐Griffin and Wing, 2016). Its counterpart in *Yersinia*, RovA, has a similar role in gene regulation and both belong to the ancient family of MarR‐like wHTH proteins (Heroven et al., 2004; Ellison and Miller, 2006; Lawrenz and Miller, 2007; Wang *et al*., 2014).

Many of the genes that are regulated by SlyA or RovA are A + T‐rich, horizontally‐acquired virulence genes that are silenced by H‐NS (Navarre *et al*., 2005; Colgan *et al*., 2016). The antiquity of these MarR‐like proteins and the simplistic nature of the genetic switches involving H‐NS antagonism suggest that they are part of a very early form of transcription control that does not have to rely on physical contact between the TF and RNA polymerase. Instead, the positively acting TF achieves its goal by removal of H‐NS or by repositioning H‐NS, permitting transcription to be initiated. In other words, these proteins operate as anti‐repressors rather than as conventional TFs (Perez *et al*., 2008).

The use of indirect readout by the MarR‐like proteins liberates them from the need to observe strictly the base sequence of their DNA targets. Instead, they are guided by DNA shape, something that can be set and reset by environmental conditions. The result is, potentially, an exquisitely versatile gene regulatory mechanism that can be adapted quickly to encompass new genes that have been acquired by horizontal transfer. With the basic on‐and‐off elements of the switch provided by the wHTH protein and H‐NS, respectively, additional specificity can be imposed by signal‐controlled TFs operating through a DNA‐sequence‐dependent direct readout mechanism. The pH‐ and osmotic‐stress‐modulated OmpR protein, with its wHTH DNA binding motif, seems to lie at the nexus of the DNA‐sequence‐dependent and DNA‐shape‐dependent worlds, combining features of both. Interestingly, several SlyA target genes are also controlled by OmpR, showing that the potential for the integration of the two regulators has been realised in several instances (e.g., the virulence genes of *Salmonella)* (Feng *et al*., 2004; Linehan *et al*., 2005).

Among the genes that are depressed by ToxR in *V. cholerae* is *leuO*, encoding the LysR‐like wHTH DNA binding protein LeuO (Bina *et al*., 2013, 2016). LysR‐type transcription factors (LTTRs) have very weak consensus DNA binding site sequences: T‐N_11_‐A, where N is any base. They also represent the largest family of TFs in bacteria (Schell, 1993; Alanazi *et al*., 2013). LeuO is an H‐NS antagonist that regulates positively genes that contribute to bile and acid resistance in *V. cholerae* (Ante *et al*., 2015a, 2015b). In *Salmonella*, the *leuO* gene is best expressed when the bacterium is grown in a medium that mimics the conditions found in a macrophage vacuole (Dillon *et al*., 2012). Here, LeuO controls a large regulon of genes, including virulence genes, many of which are silenced by H‐NS (Dillon *et al*., 2012; Cordero‐Alba and Ramos‐Morales, 2014). The *leuO* gene is regulated positively by its own product and, at least in *E. coli*, by another wHTH protein called LrhA (Breddermann and Schnetz, 2017). The *leuO* gene is located within an unusual regulatory cascade that involves transcription activation by locally generated changes in DNA negative supercoiling: a promoter relay (Fang and Wu, 1998; Dorman and Dorman, 2016). The LeuO protein relieves H‐NS‐mediated repression and cooperates with DNA topological dynamism in this relay to regulate transcription of genes involved in branched chain amino acid synthesis (Chen *et al*., 2003). This intriguing blend of *cis*‐ and *trans*‐acting regulators may hint at gene control mechanisms that predate the contributions of direct‐readout‐dependent TFs. The SpvR TF is a LysR‐like protein that is encoded by the principal virulence plasmid of non‐typhoidal strains of *S. enterica*. It regulates the *spv* operon whose products are secreted by the SPI2 secretion apparatus and alter host cell cytoskeletons (Guiney and Fierer, 2011). The *spv* operon displays sensitivity of changes in DNA supercoiling and is silenced by H‐NS (O'Byrne and Dorman, 1994; Sheehan and Dorman, 1998). Its location on a large and mobilisable plasmid illustrates the importance of horizontal gene transfer, H‐NS‐mediated silencing and sensitivity to DNA topology to the assembly and control of virulence gene clusters in pathogenic organisms.

## Concluding remarks

Variable DNA topology is, perhaps, an under‐appreciated contributor to the collective control of transcription in bacteria of all kinds, including pathogens (Dorman *et al*., 2016). Bacteria strive to control DNA supercoiling homeostatically, but the supercoiling set points change from one stage of growth to another (Conter *et al*., 1997) and as a result of environmental stresses such as acid stress (Bang *et al*., 2002), osmotic stress (Higgins *et al*., 1978), thermal stress (Goldstein and Drlica, 1984), etc. The influence of DNA supercoiling on transcription (and *vice versa*) is well established, but the potential for the changing transcriptional landscape to modulate the *binding* of proteins is sometimes overlooked in studies of gene control. Here we have emphasised the connection between DNA supercoiling and the binding to DNA of wHTH TFs. The principles that have been outlined concerning the impact of altered DNA twist on major and minor groove width, especially in A + T‐rich DNA illustrate the potential of DNA supercoiling to act allosterically on TF function (Fig. 3). The link between changes in DNA twist and adjustments to the minor and major groove width was established some time ago (Vologodskii and Cozzarelli, 1994). We suggest that these adjustments play a role in modulating the binding of proteins to the DNA. In the case of wHTH‐dependent DNA binding, the need to engage two DNA grooves of variable width provides a mechanism to enhance or to limit TF binding to target sequences that meet the requirements of the protein on base sequence criteria alone. Put crudely, this is an example of indirect readout trumping direct readout. It is an opportunity for DNA supercoiling, which is set and reset in response to the global physiological state of the cell, to influence a process that is otherwise driven by protein recognition of DNA sequence, an invariant feature of the DNA target. Is this mechanism unique to wHTH‐dependent proteins? No. Proteins relying on HTH motifs are also sensitive to variation in DNA groove width and many virulence genes are controlled by members of the large HTH‐dependent TF super‐family. The nucleoid‐associated protein Fis (Factor for Inversion Stimulation) relies on an HTH motif for DNA binding and it binds as a dimer. Docking the Fis dimer successfully to its target involves an induced fit mechanism in which minor groove compression accompanied by bending of the target DNA accommodates the side‐by‐side protein monomers (Stella *et al*., 2010). Fis binding is influenced strongly by indirect readout and minor groove conformation (Hancock *et al*., 2016). Changing the supercoiling of the DNA also exerts an effect: Fis prefers DNA that is negatively supercoiled, a requirement that is easily met during the early stages of logarithmic growth when Fis is abundant. As the cell runs out of energy and DNA relaxes, Fis binding declines (and so does Fis production) (Schneider *et al*., 1997; Cameron and Dorman, 2012). The sensitivity of DNA gyrase to the ATP/ADP ratio of the cell links DNA topology intimately to bacterial metabolism, which is in turn linked to the composition of the environment of the microbe (Hsieh *et al*., 1991a, 1991b; Van Workum *et al*., 1996). In this way, the environment can modulate in important ways the interactions of TFs of all kinds with their gene targets, adding a regulation coordination function to the genetic‐information‐carrying function of DNA.

## References

[emi13838-bib-0001] Abe, N. , Dror, I. , Yang, L. , Slattery, M. , Zhou, T. , Bussemaker, H.J. , Rohs, R. , and Mann, R.S. (2015) Deconvolving the recognition of DNA shape from sequence. Cell 161: 307–318. 25843630 10.1016/j.cell.2015.02.008 PMC4422406

[emi13838-bib-0002] Ahmer, B.M. , van Reeuwijk, J. , Watson, P.R. , Wallis, T.S. , and Heffron, F. (1999) *Salmonella* SirA is a global regulator of genes mediating enteropathogenesis. Mol Microbiol 31: 971–982. 10048039 10.1046/j.1365-2958.1999.01244.x

[emi13838-bib-0003] Alanazi, A.M. , Neidle, E.L. , and Momany, C. (2013) The DNA‐binding domain of BenM reveals the structural basis for the recognition of a T‐N_11_‐A sequence motif by LysR‐type transcriptional regulators. Acta Crystallogr D Biol Crystallogr 69: 1995–2007. 24100318 10.1107/S0907444913017320

[emi13838-bib-0004] Ali, S.S. , Whitney, J.C. , Stevenson, J. , Robinson, H. , Howell, P.L. , and Navarre, W.W. (2013) Structural insights into the regulation of foreign genes in *Salmonella* by the Hha/H‐NS complex. J Biol Chem 288: 13356–13369. 23515315 10.1074/jbc.M113.455378 PMC3650374

[emi13838-bib-0005] Ali, S.S. , Soo, J. , Rao, C. , Leung, A.S. , Ngai, D.H. , Ensminger, A.W. , and Navarre, W.W. (2014) Silencing by H‐NS potentiated the evolution of *Salmonella* . PLoS Pathog 10: e1004500. 25375226 10.1371/journal.ppat.1004500 PMC4223078

[emi13838-bib-0006] Ante, V.M. , Bina, X.R. , and Bina, J.E. (2015a) The LysR‐type regulator LeuO regulates the acid tolerance response in *Vibrio cholerae* . Microbiology 161: 2434–2443. 26424466 10.1099/mic.0.000194 PMC4811655

[emi13838-bib-0007] Ante, V.M. , Bina, X.R. , Howard, M.F. , Sayeed, S. , Taylor, D.L. , and Bina, J.E. (2015b) *Vibrio cholerae leuO* transcription is positively regulated by ToxR and contributes to bile resistance. J Bacteriol 197: 3499–3510. 26303831 10.1128/JB.00419-15 PMC4621094

[emi13838-bib-0008] Ayala, J.C. , Wang, H. , Silva, A.J. , and Benitez, J.A. (2015) Repression by H‐NS of genes required for the biosynthesis of the *Vibrio cholerae* biofilm matrix is modulated by the second messenger cyclic diguanylic acid. Mol Microbiol 97: 630–645. 25982817 10.1111/mmi.13058 PMC4617317

[emi13838-bib-0009] Ayala, J.C. , Silva, A.J. , and Benitez, J.A. (2017) H‐NS: an overarching regulator of the *Vibrio cholerae* life cycle. Res Microbiol 168: 16–25. 27492955 10.1016/j.resmic.2016.07.007 PMC5241179

[emi13838-bib-0010] Bang, I.S. , Audia, J.P. , Park, Y.K. , and Foster, J.W. (2002) Autoinduction of the *ompR* response regulator by acid shock and control of the *Salmonella enterica* acid tolerance response. Mol Microbiol 44: 1235–1250. 12068808 10.1046/j.1365-2958.2002.02937.x

[emi13838-bib-0011] Bajaj, V. , Hwang, C. , and Lee, C.A. (1995) *hilA* is a novel *ompR/toxR* family member that activates the expression of *Salmonella typhimurium* invasion genes. Mol Microbiol 18: 715–727. 8817493 10.1111/j.1365-2958.1995.mmi_18040715.x

[emi13838-bib-0012] Baños, R.C. , Pons, J.I. , Madrid, C. , and Juárez, A. (2008) A global modulatory role for the *Yersinia enterocolitica* H‐NS protein. Microbiology 154: 1281–1289. 18451036 10.1099/mic.0.2007/015610-0

[emi13838-bib-0013] Bauer, W.R. , Crick, F.H.C. , and White, J.H. (1980) Supercoiled DNA. Sci Am 243: 100–113. 6256851

[emi13838-bib-0014] Beloin, C. , and Dorman, C.J. (2003) An extended role for the nucleoid structuring protein H‐NS in the virulence gene regulatory cascade of *Shigella flexneri* . Mol Microbiol 47: 825–838. 12535079 10.1046/j.1365-2958.2003.03347.x

[emi13838-bib-0015] Bina, X.R. , Taylor, D.L. , Vikram, A. , Ante, V.M. , and Bina, J.E. (2013) *Vibrio cholerae* ToxR downregulates virulence factor production in response to cyclo(Phe‐Pro). MBio 4: e00366–13. 10.1128/mBio.00366-13 PMC3760244 23982069

[emi13838-bib-0016] Bina, X.R. , Howard, M.F. , Ante, V.M. , and Bina, J.E. (2016) *Vibrio cholerae* LeuO links the ToxR regulon to expression of lipid A remodeling genes. Infect Immun 84: 3161–3171. 27550934 10.1128/IAI.00445-16 PMC5067749

[emi13838-bib-0017] Bliven, K.A. , and Maurelli, A.T. (2016) Evolution of bacterial pathogens within the human host. Microbiol Spectr 4: 78. 10.1128/microbiolspec.VMBF-0017-2015 PMC4804625 26999399

[emi13838-bib-0018] Breddermann, H. , and Schnetz, K. (2017) Activation of *leuO* by LrhA in *Escherichia coli* . Mol Microbiol 104: 664–676. 28252809 10.1111/mmi.13656

[emi13838-bib-0019] Brennan, R.G. (1993) The winged‐helix DNA‐binding motif: another helix‐turn‐helix takeoff. Cell 74: 773–776. 8374950 10.1016/0092-8674(93)90456-z

[emi13838-bib-0020] Brennan, R.G. , and Matthews, B.W. (1989) Structural basis of DNA‐protein recognition. Trends Biochem Sci 14: 286–290. 2672451 10.1016/0968-0004(89)90066-2

[emi13838-bib-0021] Browning, D.F. , and Busby, S.J.W. (2016) Local and global regulation of transcription initiation in bacteria. Nat Rev Microbiol 14: 638–650. 27498839 10.1038/nrmicro.2016.103

[emi13838-bib-0022] Cai, S.J. , and Inouye, M. (2002) EnvZ‐OmpR interaction and osmoregulation in *Escherichia coli* . J Biol Chem 277: 24155–24161. 11973328 10.1074/jbc.M110715200

[emi13838-bib-0023] Cameron, A.D. , and Dorman, C.J. (2012) A fundamental regulatory mechanism operating through OmpR and DNA topology controls expression of *Salmonella* pathogenicity islands SPI‐1 and SPI‐2. PLoS Genet 8: e1002615. 22457642 10.1371/journal.pgen.1002615 PMC3310775

[emi13838-bib-0024] Carroll, R.K. , Liao, X. , Morgan, L.K. , Cicirelli, E.M. , Li, Y. , Sheng, W. , Feng, X. , and Kenney, L.J. (2009) Structural and functional analysis of the C‐terminal DNA binding domain of the *Salmonella typhimurium* SPI‐2 response regulator SsrB. J Biol Chem 284: 12008–12019. 19126546 10.1074/jbc.M806261200 PMC2673270

[emi13838-bib-0025] Casadesús, J. (2016) Bacterial DNA methylation and methylomes. Adv Exp Med Biol 945: 35–61. 27826834 10.1007/978-3-319-43624-1_3

[emi13838-bib-0026] Cerdan, R. , Bloch, V. , Yang, Y. , Bertin, P. , Dumas, C. , Rimsky, S. , Kochoyan, M. , and Arold, S.T. (2003) Crystal structure of the N‐terminal dimerisation domain of VicH, the H‐NS‐like protein of *Vibrio cholerae* . J Mol Biol 334: 179–185. 14607110 10.1016/j.jmb.2003.09.051

[emi13838-bib-0027] Chen, B. , de Crombrugghe, B. , Anderson, W.B. , Gottesman, M.E. , Pastan, I. , and Perlman, R.L. (1971) On the mechanism of action of *lac* repressor. Nat New Biol 233: 67–70. 4330295 10.1038/newbio233067a0

[emi13838-bib-0028] Chen, C.C. , Ghole, M. , Majumder, A. , Wang, Z. , Chandana, S. , and Wu, H.Y. (2003) LeuO‐mediated transcriptional derepression. J Biol Chem 278: 38094–38103. 12871947 10.1074/jbc.M300461200

[emi13838-bib-0029] Chiu, T.‐P. , Yang, L. , Zhou, T. , Main, B.J. , Parker, S.C.J. , Nuzhdin, S.V. , Tullius, T.D. , and Rohs, R. (2014) GBshape: a genome browser database for DNA shape annotations. Nucleic Acids Res 43: D103–D109. 25326329 10.1093/nar/gku977 PMC4384032

[emi13838-bib-0030] Colgan, A.M. , Kröger, C. , Diard, M. , Hardt, W.D. , Puente, J.L. , Sivasankaran, S.K. , Hokamp, K. , and Hinton, J.C. (2016) The impact of 18 ancestral and horizontally‐acquired regulatory proteins upon the transcriptome and sRNA landscape of *Salmonella enterica* serovar Typhimurium. PLoS Genet 12: e1006258. 27564394 10.1371/journal.pgen.1006258 PMC5001712

[emi13838-bib-0031] Conter, A. , Menchon, C. , and Gutierrez, C. (1997) Role of DNA supercoiling and *rpoS* sigma factor in the osmotic and growth phase‐dependent induction of the gene *osmE* of *Escherichia coli* K12. J Mol Biol 273: 75–83. 9367747 10.1006/jmbi.1997.1308

[emi13838-bib-0032] Corbett, D. , Bennett, H.J. , Askar, H. , Green, J. , and Roberts, I.S. (2007) SlyA and H‐NS regulate transcription of the *Escherichia coli* K5 capsule gene cluster, and expression of *slyA* in *Escherichia coli* is temperature‐dependent, positively autoregulated, and independent of H‐NS. J Biol Chem 282: 33326–33335. 17827501 10.1074/jbc.M703465200

[emi13838-bib-0033] Cordero‐Alba, M. , and Ramos‐Morales, F. (2014) Patterns of expression and translocation of the ubiquitin ligase SirP in *Salmonella enterica* serovar Typhimurium. J Bacteriol 196: 3912–3922. 25182488 10.1128/JB.02158-14 PMC4248824

[emi13838-bib-0034] Dame, R.T. , Wyman, C. , and Goosen, N. (2001) Structural basis for preferential binding of H‐NS to curved DNA. Biochimie 83: 231–234. 11278073 10.1016/s0300-9084(00)01213-x

[emi13838-bib-0035] Dame, R.T. , Wyman, C. , Wurm, R. , Wagner, R. , and Goosen, N. (2002) Structural basis for H‐NS‐mediated trapping of RNA polymerase in the open initiation complex at the *rrnB* P1. J Biol Chem 277: 2146–2150. 11714691 10.1074/jbc.C100603200

[emi13838-bib-0036] Delgado, J. , Forst, S. , Harlocker, S. , and Inouye, M. (1993) Identification of a phosphorylation site and functional analysis of conserved aspartic acid residues of OmpR, a transcriptional activator for *ompF* and *ompC* in *Escherichia coli* . Mol Microbiol 10: 1037–1047. 7934854 10.1111/j.1365-2958.1993.tb00974.x

[emi13838-bib-0037] Dillon, S.C. , and Dorman, C.J. (2010) Bacterial nucleoid‐associated proteins, nucleoid structure and gene expression. Nat Rev Microbiol 8: 185–195. 20140026 10.1038/nrmicro2261

[emi13838-bib-0038] Dillon, S.C. , Espinosa, E. , Hokamp, K. , Ussery, D.W. , Casadesús, J. , and Dorman, C.J. (2012) LeuO is a global regulator of gene expression in *Salmonella enterica* serovar Typhimurium. Mol Microbiol 85: 1072–1089. 22804842 10.1111/j.1365-2958.2012.08162.x

[emi13838-bib-0039] Dolan, K.T. , Duguid, E.M. , and He, C. (2011) Crystal structures of SlyA protein, a master virulence regulator of *Salmonella*, in free and DNA‐bound states. J Biol Chem 286: 22178–22185. 21550983 10.1074/jbc.M111.245258 PMC3121362

[emi13838-bib-0040] Dorman, C.J. (2004) H‐NS: a universal regulator for a dynamic genome. Nat Rev Microbiol 2: 391–400. 15100692 10.1038/nrmicro883

[emi13838-bib-0041] Dorman, C.J. (2007) H‐NS, the genome sentinel. Nat Rev Microbiol 5: 157–161. 17191074 10.1038/nrmicro1598

[emi13838-bib-0042] Dorman, C.J. (2009) Regulatory integration of horizontally‐transferred genes in bacteria. Front Biosci (Landmark Ed) 14: 4103–4112. 19273337 10.2741/3515

[emi13838-bib-0043] Dorman, C.J. (2015) Integrating small molecule signalling and H‐NS antagonism in *Vibrio cholerae*, a bacterium with two chromosomes. Mol Microbiol 97: 612–615. 25988304 10.1111/mmi.13063

[emi13838-bib-0044] Dorman, C.J. , and Kane, K.A. (2009) DNA bridging and antibridging: a role for bacterial nucleoid‐associated proteins in regulating the expression of laterally acquired genes. FEMS Microbiol Rev 33: 587–592. 19207739 10.1111/j.1574-6976.2008.00155.x

[emi13838-bib-0045] Dorman, C.J. , and Dorman, M.J. (2016) DNA supercoiling is a fundamental regulatory principle in the control of bacterial gene expression. Biophys Rev 8: 209–220. 28510224 10.1007/s12551-016-0205-y PMC5425793

[emi13838-bib-0046] Dorman, C.J. , Colgan, A. , and Dorman, M.J. (2016) Bacterial pathogen gene regulation: a DNA‐structure‐centred view of a protein‐dominated domain. Clin Sci 130: 1165–1177. 27252403 10.1042/CS20160024

[emi13838-bib-0047] Eichelberg, K. , and Galán, J.E. (1999) Differential regulation of *Salmonella typhimurium* type III secreted proteins by pathogenicity island 1 (SPI‐1)‐encoded transcriptional activators InvF and HilA. Infect Immun 67: 4099–4105. 10417179 10.1128/iai.67.8.4099-4105.1999 PMC96710

[emi13838-bib-0048] Ellison, D.W. , and Miller, V.L. (2006) Regulation of virulence by members of the MarR/SlyA family. Curr Opin Microbiol 9: 153–159. 16529980 10.1016/j.mib.2006.02.003

[emi13838-bib-0049] Erhardt, M. , and Dersch, P. (2015) Regulatory principles governing *Salmonella* and *Yersinia* virulence. Front Microbiol 6: 949. 26441883 10.3389/fmicb.2015.00949 PMC4563271

[emi13838-bib-0050] Fahlen, T.F. , Wilson, R.L. , Boddicker, J.D. , and Jones, B.D. (2001) Hha is a negative modulator of transcription of *hilA*, the *Salmonella enterica* serovar Typhimurium invasion gene transcriptional activator. J Bacteriol 183: 6620–6629. 11673432 10.1128/JB.183.22.6620-6629.2001 PMC95493

[emi13838-bib-0051] Fang, M. , and Wu, H.Y. (1998) A promoter relay mechanism for sequential gene activation. J Bacteriol 180: 626–633. 9457867 10.1128/jb.180.3.626-633.1998 PMC106931

[emi13838-bib-0052] Fass, E. , and Groisman, E.A. (2009) Control of *Salmonella* pathogenicity island‐2 gene expression. Curr Opin Microbiol 12: 199–204. 19264535 10.1016/j.mib.2009.01.004 PMC2805070

[emi13838-bib-0053] Feng, X. , Oropeza, R. , and Kenney, L.J. (2003) Dual regulation by phospho‐OmpR of *ssrA/B* gene expression in *Salmonella* pathogenicity island 2. Mol Microbiol 48: 1131–1143. 12753201 10.1046/j.1365-2958.2003.03502.x

[emi13838-bib-0054] Feng, X. , Walthers, D. , Oropeza, R. , and Kenney, L.J. (2004) The response regulator SsrB activates transcription and binds to a region overlapping OmpR binding sites at *Salmonella* pathogenicity island 2. Mol Microbiol 54: 823–835. 15491370 10.1111/j.1365-2958.2004.04317.x

[emi13838-bib-0055] Foo, Y.H. , Gao, Y. , Zhang, H. , and Kenney, L.J. (2015) Cytoplasmic sensing by the inner membrane histidine kinase EnvZ. Prog Biophys Mol Biol 118: 119–129. 25937465 10.1016/j.pbiomolbio.2015.04.005 PMC5080436

[emi13838-bib-0056] Gellert, M. , Mizuuchi, K. , Odea, M.H. , and Nash, H.A. (1976) DNA gyrase: enzyme that introduces superhelical turns into DNA. Proc Natl Acad Sci USA 73: 3872–3876. 186775 10.1073/pnas.73.11.3872 PMC431247

[emi13838-bib-0057] Goldstein, E. , and Drlica, K. (1984) Regulation of bacterial DNA supercoiling: plasmid linking numbers vary with growth temperature. Proc Natl Acad Sci USA 81: 4046–4050. 6377307 10.1073/pnas.81.13.4046 PMC345365

[emi13838-bib-0058] Gordân, R. , Shen, N. , Dror, I. , Zhou, T. , Horton, J. , Rohs, R. , and Bulyk, M.L. (2013) Genomic regions flanking E‐box binding sites influence DNA binding specificity of bHLH transcription factors through DNA shape. Cell Rep 3: 1093–1104. 23562153 10.1016/j.celrep.2013.03.014 PMC3640701

[emi13838-bib-0059] Guiney, D.G. , and Fierer, J. (2011) The role of the *spv* genes in *Salmonella* pathogenesis. Front Microbiol 2: 129. 21716657 10.3389/fmicb.2011.00129 PMC3117207

[emi13838-bib-0060] Haas, B.L. , Matson, J.S. , DiRita, V.J. , and Biteen, J.S. (2015) Single‐molecule tracking in live *Vibrio cholerae* reveals that ToxR recruits the membrane‐bound virulence regulator TcpP to the ToxT promoter. Mol Microbiol 96: 4–13. 25318589 10.1111/mmi.12834 PMC6025817

[emi13838-bib-0061] Hancock, S.P. , Stella, S. , Cascio, D. , and Johnson, R.C. (2016) DNA Sequence determinants controlling affinity, stability and shape of DNA complexes bound by the nucleoid protein Fis. PLoS One 11: e0150189. 26959646 10.1371/journal.pone.0150189 PMC4784862

[emi13838-bib-0062] Harrison, S.C. , and Aggarwal, A.K. (1990) DNA recognition by proteins with the helix‐turn‐helix motif. Annu Rev Biochem 59: 933–969. 2197994 10.1146/annurev.bi.59.070190.004441

[emi13838-bib-0063] Hazen, T.H. , Pan, L. , Gu, J.D. , and Sobecky, P.A. (2010) The contribution of mobile genetic elements to the evolution and ecology of Vibrios. FEMS Microbiol Ecol 74: 485–499. 20662928 10.1111/j.1574-6941.2010.00937.x

[emi13838-bib-0064] Heroven, A.K. , Nagel, G. , Tran, H.J. , Parr, S. , and Dersch, P. (2004) RovA is autoregulated and antagonizes H‐NS‐mediated silencing of invasin and *rovA* expression in *Yersinia pseudotuberculosis* . Mol Microbiol 53: 871–888. 15255899 10.1111/j.1365-2958.2004.04162.x

[emi13838-bib-0065] Higgins, D.E. , and DiRita, V.J. (1994) Transcriptional control of *toxT*, a regulatory gene in the ToxR regulon of *Vibrio cholerae* . Mol Microbiol 14: 17–29. 7830555 10.1111/j.1365-2958.1994.tb01263.x

[emi13838-bib-0066] Higgins, N.P. , Peebles, C.L. , Sugino, A. , and Cozzarelli, N.R. (1978) Purification of subunits of *Escherichia coli* DNA gyrase and reconstitution of enzymatic activity. Proc Natl Acad Sci USA 75: 1773–1777. 347446 10.1073/pnas.75.4.1773 PMC392422

[emi13838-bib-0067] Hromockyj, A.E. , Tucker, S.C. , and Maurelli, A.T. (2002) Temperature regulation of *Shigella* virulence: identification of the repressor gene virR, an analogue of *hns*, and partial complementation by tyrosyl transfer RNA ( ${\text{tRNA}}_{1}^{\text{Tyr}}$). Mol Microbiol 6: 2113–2124. 10.1111/j.1365-2958.1992.tb01385.x 1406252

[emi13838-bib-0068] Hsieh, L.S. , Burger, R.M. , and Drlica, K. (1991a) Bacterial DNA supercoiling and [ATP]/[ADP]. Changes associated with a transition to anaerobic growth. J Mol Biol 219: 443–450. 1646892 10.1016/0022-2836(91)90185-9

[emi13838-bib-0069] Hsieh, L.S. , Rouvière‐Yaniv, J. , and Drlica, K. (1991b) Bacterial DNA supercoiling and [ATP]/[ADP] ratio: changes associated with salt shock. J Bacteriol 173: 3914–3917. 1646791 10.1128/jb.173.12.3914-3917.1991 PMC208027

[emi13838-bib-0070] Hüttener, M. , Paytubi, S. , and Juárez, A. (2015) Success in incorporating horizontally transferred genes: the H‐NS protein. Trends Microbiol 23: 67–69. 25560233 10.1016/j.tim.2014.12.009

[emi13838-bib-0071] Johnson, A. , Meyer, B.J. , and Ptashne, M. (1978) Mechanism of action of the cro protein of bacteriophage lambda. Proc Natl Acad Sci USA 75: 1783–1787. 273909 10.1073/pnas.75.4.1783 PMC392424

[emi13838-bib-0072] Joshi, R. , Passner, J.M. , Rohs, R. , Jain, R. , Sosinsky, A. , Crickmore, M.A. , *et al* (2007) Functional specificity of a Hox protein mediated by the recognition of minor groove structure. Cell 131: 530–543. 17981120 10.1016/j.cell.2007.09.024 PMC2709780

[emi13838-bib-0073] Kane, K.A. , and Dorman, C.J. (2011) Rational design of an artificial genetic switch: co‐option of the H‐NS‐repressed *proU* operon by the VirB virulence master regulator. J Bacteriol 193: 5950–5960. 21873493 10.1128/JB.05557-11 PMC3194901

[emi13838-bib-0074] Kazi, M.I. , Conrado, A.R. , Mey, A.R. , Payne, S.M. , and Davies, B.W. (2016) ToxR antagonizes H‐NS regulation of horizontally acquired genes to drive host colonization. PLoS Pathog 12: e1005570. 27070545 10.1371/journal.ppat.1005570 PMC4829181

[emi13838-bib-0075] Kenney, L.J. (2002) Structure/function relationships in OmpR and other winged‐helix transcription factors. Curr Opin Microbiol 5: 135–341. 11934608 10.1016/s1369-5274(02)00310-7

[emi13838-bib-0076] Krukonis, E.S. , and DiRita, V. (2003) DNA binding and ToxR responsiveness by the wing domain of TcpP, an activator of virulence gene expression in *Vibrio cholerae* . Mol Cell 12: 157–165. 12887901 10.1016/s1097-2765(03)00222-3

[emi13838-bib-0077] Lang, B. , Blot, N. , Bouffartigues, E. , Buckle, M. , Geertz, M. , Gualerzi, C.O. , *et al* (2007) High‐affinity DNA binding sites for H‐NS provide a molecular basis for selective silencing within proteobacterial genomes. Nucleic Acids Res 35: 6330–6337. 17881364 10.1093/nar/gkm712 PMC2094087

[emi13838-bib-0078] Lawrenz, M.B. , and Miller, V.L. (2007) Comparative analysis of the regulation of *rovA* from the pathogenic yersiniae. J Bacteriol 189: 5963–5975. 17573476 10.1128/JB.00528-07 PMC1952055

[emi13838-bib-0079] Linehan, S.A. , Rytkönen, A. , Yu, X.J. , Liu, M. , and Holden, D.W. (2005) SlyA regulates function of *Salmonella* pathogenicity island 2 (SPI‐2) and expression of SPI‐2‐associated genes. Infect Immun 73: 4354–4362. 15972530 10.1128/IAI.73.7.4354-4362.2005 PMC1168564

[emi13838-bib-0080] Lithgow, J.K. , Haider, F. , Roberts, I.S. , and Green, J. (2007) Alternate SlyA and H‐NS nucleoprotein complexes control *hlyE* expression in *Escherichia coli* K‐12. Mol Microbiol 66: 685–698. 17892462 10.1111/j.1365-2958.2007.05950.x PMC2156107

[emi13838-bib-0081] Liu, L.F. , and Wang, J.C. (1987) Supercoiling of the DNA template during transcription. Proc Natl Acad Sci USA 84: 7024–7027. 2823250 10.1073/pnas.84.20.7024 PMC299221

[emi13838-bib-0082] Lostroh, C.P. , Bajaj, V. , and Lee, C.A. (2000) The *cis* requirements for transcriptional activation by HilA, a virulence determinant encoded on SPI‐1. Mol Microbiol 37: 300–315. 10931326 10.1046/j.1365-2958.2000.01991.x

[emi13838-bib-0083] Lucchini, S. , Rowley, G. , Goldberg, M.D. , Hurd, D. , Harrison, M. , and Hinton, J.C. (2006) H‐NS mediates the silencing of laterally acquired genes in bacteria. PLoS Pathog 2: e81. 16933988 10.1371/journal.ppat.0020081 PMC1550270

[emi13838-bib-0084] Martínez‐Hackert, E. , and Stock, A.M. (1997) Structural relationships in the OmpR family of winged‐helix transcription factors. J Mol Biol 269: 301–312. 9199401 10.1006/jmbi.1997.1065

[emi13838-bib-0085] Main‐Hester, K.L. , Colpitts, K.M. , Thomas, G.A. , Fang, F.C. , and Libby, S.J. (2008) Coordinate regulation of *Salmonella* pathogenicity island 1 (SPI1) and SPI4 in *Salmonella enterica* serovar Typhimurium. Infect Immun 76: 1024–1035. 18160484 10.1128/IAI.01224-07 PMC2258849

[emi13838-bib-0086] Mathelier, A. , Xin, B. , Chiu, T.P. , Yang, L. , Rohs, R. , and Wasserman, W.W. (2016) DNA shape features improve transcription factor binding site predictions *in vivo* . Cell Syst 3: 278–286. 27546793 10.1016/j.cels.2016.07.001 PMC5042832

[emi13838-bib-0087] Mendieta, J. , Pérez‐Lago, L. , Salas, M. , and Camacho, A. (2007) DNA sequence‐specific recognition by a transcriptional regulator requires indirect readout of A‐tracts. Nucleic Acids Res 35: 3252–3261. 17452358 10.1093/nar/gkm180 PMC1904284

[emi13838-bib-0088] Miller, V.L. , Taylor, R.K. , and Mekalanos, J.J. (1987) Cholera toxin transcriptional activator *toxR* is a transmembrane DNA binding protein. Cell 48: 271–279. 3802195 10.1016/0092-8674(87)90430-2

[emi13838-bib-0089] Navarre, W.W. (2016) The impact of gene silencing on horizontal gene transfer and bacterial evolution. Adv Microb Physiol 69: 157–186. 27720010 10.1016/bs.ampbs.2016.07.004

[emi13838-bib-0090] Navarre, W.W. , Halsey, T.A. , Walthers, D. , Frye, J. , McClelland, M. , Potter, J.L. , *et al* (2005) Co‐regulation of *Salmonella enterica* genes required for virulence and resistance to antimicrobial peptides by SlyA and PhoP/PhoQ. Mol Microbiol 56: 492–508. 15813739 10.1111/j.1365-2958.2005.04553.x

[emi13838-bib-0091] Nye, M.B. , Pfau, J.D. , Skorupski, K. , and Taylor, R.K. (2000) *Vibrio cholerae* H‐NS silences virulence gene expression at multiple steps in the ToxR regulatory cascade. J Bacteriol 182: 4295–4303. 10894740 10.1128/jb.182.15.4295-4303.2000 PMC101945

[emi13838-bib-0092] O'Byrne, C.P. , and Dorman, C.J. (1994) Transcription of the *Salmonella typhimurium spv* virulence locus is regulated negatively by the nucleoid‐associated protein H‐NS. FEMS Microbiol Lett 121: 99–105. 8082832 10.1111/j.1574-6968.1994.tb07082.x

[emi13838-bib-0093] Ochman, H. , Lawrence, J.G. , and Groisman, E.A. (2000) Lateral gene transfer and the nature of bacterial innovation. Nature 405: 299–304. 10830951 10.1038/35012500

[emi13838-bib-0094] Parsot, C. , and Mekalanos, J.J. (1992) Structural analysis of the *acfA* and *acfD* genes of *Vibrio* cholerae: effects of DNA topology and transcriptional activators on expression. J Bacteriol 174: 5211–5218. 1644747 10.1128/jb.174.16.5211-5218.1992 PMC206354

[emi13838-bib-0095] Perez, J.C. , Latifi, T. , and Groisman, E.A. (2008) Overcoming H‐NS‐mediated transcriptional silencing of horizontally acquired genes by the PhoP and SlyA proteins in *Salmonella enterica* . J Biol Chem 283: 10773–10783. 18270203 10.1074/jbc.M709843200 PMC2447644

[emi13838-bib-0096] Porter, M.E. , and Dorman, C.J. (1994) A role for H‐NS in the thermo‐osmotic regulation of virulence gene expression in *Shigella flexneri* . J Bacteriol 176: 4187–4191. 8021202 10.1128/jb.176.13.4187-4191.1994 PMC205622

[emi13838-bib-0097] Pratt, L.A. , Hsing, W. , Gibson, K.E. , and Silhavy, T.J. (1996) From acids to *osmZ*: multiple factors influence synthesis of the OmpF and OmpC porins in *Escherichia coli* . Mol Microbiol 20: 911–917. 8809744 10.1111/j.1365-2958.1996.tb02532.x

[emi13838-bib-0098] Prieto, A. , Urcola, I. , Blanco, J. , Dahbi, G. , Muniesa, M. , Quirós, P. , *et al* (2016) Tracking bacterial virulence: global modulators as indicators. Sci Rep 6: 25973. 27169404 10.1038/srep25973 PMC4864382

[emi13838-bib-0099] Prosseda, G. , Falconi, M. , Giangrossi, M. , Gualerzi, C.O. , Micheli, G. , and Colonna, B. (2004) The *virF* promoter in *Shigella*: more than just a curved DNA stretch. Mol Microbiol 51: 523–537. 14756791 10.1046/j.1365-2958.2003.03848.x

[emi13838-bib-0100] Queiroz, M.H. , Madrid, C. , Paytubi, S. , Balsalobre, C. , and Juárez, A. (2011) Integration host factor alleviates H‐NS silencing of the *Salmonella enterica* serovar Typhimurium master regulator of SPI1, *hilA* . Microbiology 157: 2504–2514. 21680637 10.1099/mic.0.049197-0

[emi13838-bib-0101] Quinn, H.J. , Cameron, A.D. , and Dorman, C.J. (2014) Bacterial regulon evolution: distinct responses and roles for the identical OmpR proteins of *Salmonella* Typhimurium and *Escherichia coli* in the acid stress response. PLoS Genet 10: e1004215. 24603618 10.1371/journal.pgen.1004215 PMC3945435

[emi13838-bib-0102] Rhee, J.E. , Sheng, W. , Morgan, L.K. , Nolet, R. , Liao, X. , and Kenney, L.J. (2008) Amino acids important for DNA recognition by the response regulator OmpR. J Biol Chem 283: 8664–8677. 18195018 10.1074/jbc.M705550200 PMC2417188

[emi13838-bib-0103] Rhen, M. , and Dorman, C.J. (2005) Hierarchical gene regulators adapt *Salmonella enterica* to its host milieus. Int J Med Microbiol 294: 487–502. 15790293 10.1016/j.ijmm.2004.11.004

[emi13838-bib-0104] Robins, W.P. , and Mekalanos, J.J. (2014) Genomic science in understanding cholera outbreaks and evolution of *Vibrio cholerae* as a human pathogen. Curr Top Microbiol Immunol 379: 211–229. 24590676 10.1007/82_2014_366 PMC4153709

[emi13838-bib-0105] Rodriguez‐Valera, F. , Martin‐Cuadrado, A.B. , and López‐Pérez, M. (2016) Flexible genomic islands as drivers of genome evolution. Curr Opin Microbiol 31: 154–160. 27085300 10.1016/j.mib.2016.03.014

[emi13838-bib-0106] Rohs, R. , West, S.M. , Sosinsky, A. , Liu, P. , Mann, R.S. , and Honig, B. (2009) The role of DNA shape in protein‐DNA recognition. Nature 461: 1248–1253. 19865164 10.1038/nature08473 PMC2793086

[emi13838-bib-0107] Rohs, R. , Jin, X. , West, S.M. , Joshi, R. , Honig, B. , and Mann, R.S. (2010) Origins of specificity in protein‐DNA recognition. Annu Rev Biochem 79: 233–269. 20334529 10.1146/annurev-biochem-060408-091030 PMC3285485

[emi13838-bib-0108] Schell, M.A. (1993) Molecular biology of the LysR family of transcriptional regulators. Annu Rev Microbiol 47: 597–626. 8257110 10.1146/annurev.mi.47.100193.003121

[emi13838-bib-0109] Schneider, R. , Travers, A. , and Muskhelishvili, G. (1997) FIS modulates growth phase‐dependent topological transitions of DNA in *Escherichia coli* . Mol Microbiol 26: 519–530. 9402022 10.1046/j.1365-2958.1997.5951971.x

[emi13838-bib-0110] Sheehan, B.J. , and Dorman, C.J. (1998) *In vivo* analysis of the interactions of the LysR‐like regulator SpvR with the operator sequences of the *spvA* and *spvR* virulence genes of *Salmonella typhimurium* . Mol Microbiol 30: 91–105. 9786188 10.1046/j.1365-2958.1998.01041.x

[emi13838-bib-0111] Skorupski, K. , and Taylor, R.K. (1997) Control of the ToxR virulence regulon in *Vibrio cholerae* by environmental stimuli. Mol Microbiol 25: 1003–1009. 9350858 10.1046/j.1365-2958.1997.5481909.x

[emi13838-bib-0112] Slattery, M. , Zhou, T. , Yang, L. , Dantas Machado, A.C. , Gordân, R. , and Rohs, R. (2014) Absence of a simple code: how transcription factors read the genome. Trends Biochem Sci 39: 381–399. 25129887 10.1016/j.tibs.2014.07.002 PMC4149858

[emi13838-bib-0113] Steffen, N.R. , Murphy, S.D. , Tolleri, L. , Hatfield, G.W. , and Lathrop, R.H. (2002) DNA sequence and structure: direct and indirect recognition in protein‐DNA binding. Bioinformatics 18 Suppl 1: S22–S30. 12169527 10.1093/bioinformatics/18.suppl_1.s22

[emi13838-bib-0114] Steitz, T.A. , Ohlendorf, D.H. , McKay, D.B. , Anderson, W.F. , and Matthews, B.W. (1982) Structural similarity in the DNA‐binding domains of catabolite gene activator and cro repressor proteins. Proc Natl Acad Sci USA 79: 3097–3100. 6212926 10.1073/pnas.79.10.3097 PMC346360

[emi13838-bib-0115] Stella, S. , Cascio, D. , and Johnson, R.C. (2010) The shape of the DNA minor groove directs binding by the DNA‐bending protein Fis. Genes Dev 24: 814–826. 20395367 10.1101/gad.1900610 PMC2854395

[emi13838-bib-0116] Stincone, A. , Daudi, N. , Rahman, A.S. , Antczak, P. , Henderson, I. , Cole, J. , *et al* (2011) A systems biology approach sheds new light on *Escherichia coli* acid resistance. Nucleic Acids Res 39: 7512–7528. 21690099 10.1093/nar/gkr338 PMC3177180

[emi13838-bib-0117] Stoebel, D.M. , Free, A. , and Dorman, C.J. (2008) Anti‐silencing: overcoming H‐NS‐mediated repression of transcription in Gram‐negative enteric bacteria. Microbiology 154: 2533–2545. 18757787 10.1099/mic.0.2008/020693-0

[emi13838-bib-0118] Syvanen, M. (2012) Evolutionary implications of horizontal gene transfer. Annu Rev Genet 46: 341–358. 22934638 10.1146/annurev-genet-110711-155529

[emi13838-bib-0119] Tendeng, C. , Badaut, C. , Krin, E. , Gounon, P. , Ngo, S. , Danchin, A. , Rimsky, S. , and Bertin, P. (2000) Isolation and characterization of *vicH*, encoding a new pleiotropic regulator in *Vibrio cholerae* . J Bacteriol 182: 2026–2032. 10715012 10.1128/jb.182.7.2026-2032.2000 PMC101921

[emi13838-bib-0120] Thijs, I.M. , De Keersmaecker, S.C. , Fadda, A. , Engelen, K. , Zhao, H. , McClelland, M. , Marchal, K. , and Vanderleyden, J. (2007) Delineation of the *Salmonella enterica* serovar Typhimurium HilA regulon through genome‐wide location and transcript analysis. J Bacteriol 189: 4587–4596. 17483226 10.1128/JB.00178-07 PMC1913449

[emi13838-bib-0121] Tobe, T. , Yoshikawa, M. , and Sasakawa, C. (1995) Thermoregulation of *virB* transcription in *Shigella flexneri* by sensing of changes in local DNA superhelicity. J Bacteriol 177: 1094–1097. 7860590 10.1128/jb.177.4.1094-1097.1995 PMC176708

[emi13838-bib-0122] Travers, A.A. (1997) DNA‐protein interactions: IHF ‐ the master bender. Curr Biol 7: R252–R254. 9162504 10.1016/s0960-9822(06)00114-x

[emi13838-bib-0123] Van Workum, M. , van Dooren, S.J. , Oldenburg, N. , Molenaar, D. , Jensen, P.R. , Snoep, J.L. , and Westerhoff, H.V. (1996) DNA supercoiling depends on the phosphorylation potential in *Escherichia coli* . Mol Microbiol 20: 351–360. 8733233 10.1111/j.1365-2958.1996.tb02622.x

[emi13838-bib-0124] Vologodskii, A.V. , and Cozzarelli, N.R. (1994) Conformational and thermodynamic properties of supercoiled DNA. Annu Rev Biophys Biomol Struct 23: 609–643. 7919794 10.1146/annurev.bb.23.060194.003141

[emi13838-bib-0125] Waldor, M.K. , and Mekalanos, J.J. (1996) Lysogenic conversion by a filamentous phage encoding cholera toxin. Science 272: 1910–1914. 8658163 10.1126/science.272.5270.1910

[emi13838-bib-0126] Walthers, D. , Li, Y. , Liu, Y. , Anand, G. , Yan, J. , and Kenney, L.J. (2011) *Salmonella enterica* response regulator SsrB relieves H‐NS silencing by displacing H‐NS bound in polymerization mode and directly activates transcription. J Biol Chem 286: 1895–1902. 21059643 10.1074/jbc.M110.164962 PMC3023485

[emi13838-bib-0127] Wang, D. , Guo, C. , Gu, L. , and Zhang, X. (2014) Comparative study of the *marR* genes within the family Enterobacteriaceae. J Microbiol 52: 452–459. 24723108 10.1007/s12275-014-3586-2

[emi13838-bib-0128] Weatherspoon‐Griffin, N. , and Wing, H.J. (2016) Characterization of SlyA in *Shigella flexneri* identifies a novel role in virulence. Infect Immun 84: 1073–1082. 26831468 10.1128/IAI.00806-15 PMC4807491

[emi13838-bib-0129] Westermark, M. , Oscarsson, J. , Mizunoe, Y. , Urbonaviciene, J. , and Uhlin, B.E. (2000) Silencing and activation of ClyA cytotoxin expression in *Escherichia coli* . J Bacteriol 182: 6347–6357. 11053378 10.1128/jb.182.22.6347-6357.2000 PMC94780

[emi13838-bib-0130] Yang, L. , Orenstein, Y. , Jolma, A. , Yin, Y. , Taipale, J. , Shamir, R. , and Rohs, R. (2017) Transcription factor family‐specific DNA shape readout revealed by quantitative specificity models. Mol Syst Biol 13: 910. 28167566 10.15252/msb.20167238 PMC5327724

[emi13838-bib-0131] Yu, R.R. , and DiRita, V.J. (1999) Analysis of an autoregulatory loop controlling ToxT, cholera toxin, and toxin‐coregulated pilus production in *Vibrio cholerae* . J Bacteriol 181: 2584–2592. 10198025 10.1128/jb.181.8.2584-2592.1999 PMC93687

[emi13838-bib-0132] Zimmerman, S.B. (2006) Shape and compaction of *Escherichia coli* nucleoids. J Struct Biol 156: 255–261. 16697220 10.1016/j.jsb.2006.03.022

[emi13838-bib-0133] Zwir, I. , Yeo, W.S. , Shin, D. , Latifi, T. , Huang, H. , and Groisman, E.A. (2014) Bacterial nucleoid‐associated protein uncouples transcription levels from transcription timing. MBio 5: e01485–14. 25293763 10.1128/mBio.01485-14 PMC4196223

